# Empathy expectations: Trait empathy exacerbates apologetic offenders' negative reactions to non‐forgiveness

**DOI:** 10.1111/bjso.70001

**Published:** 2025-06-24

**Authors:** Blake Quinney, Michael Wenzel, Michael Thai, Tyler Okimoto, Lydia Woodyatt

**Affiliations:** ^1^ The University of Adelaide Adelaide South Australia Australia; ^2^ Flinders University Adelaide South Australia Australia; ^3^ The University of Queensland Brisbane Queensland Australia

**Keywords:** apology, empathy, forgiveness, interpersonal relationships, norms, reconciliation, transgressions

## Abstract

Victims have the prerogative to withhold forgiveness. However, offenders who apologize may believe that they have acted correctly and can feel wronged by victims who refuse to forgive in return. Indeed, apologetic offenders can perceive victims' non‐forgiveness as violating an apology‐forgiveness reciprocity norm and as a threat to their own sense of status/power, which makes offenders perceive themselves as victims, and less willing to engage in further reconciliatory behaviour. The present research investigates whether offenders' trait empathy can qualify these destructive responses to non‐forgiveness. We originally theorized that a greater capacity to empathize with victims may help offenders better understand victims' non‐forgiveness and react less negatively to it. Across three studies (combined *N* = 1000), we find evidence of the contrary—offenders who have high trait empathy tend to react more negatively to non‐forgiving victims. Our findings suggest this is because empathic offenders believe that victims should reciprocate their reparatory action with an empathic response. This presents a conundrum for repair processes that promote empathy.

## INTRODUCTION

Empathy is widely believed to help overcome barriers to reconciliation. A broad literature base touts the importance of empathy for promoting reconciliation, both as an individual attribute and as a process intervention that facilitates a caring understanding of others and promotes forgiveness (Wallis, 2014; Wohl & McGrath, 2007). Likewise, it could be reasoned that offenders' trait empathy may promote their understanding of victims' reluctance to accept their apology and forgive them. Empathic offenders may have a greater capacity to recognize the hurt caused to victims and the victims' prerogative to decide not to forgive.

For reconciliation, offenders' empathy may be important because there is evidence that if victims refuse to forgive in response to an apology, offenders can experience this forgiveness refusal as a norm violation and a disrespect or disempowerment that leads them to perceive themselves as ‘victims’ (Thai et al., 2023). As a consequence, offenders may feel resentment and disengage from the moral repair process (Jennings et al., 2016; Thai et al., 2023). Victims may also anticipate offenders' backlash to non‐forgiveness (Baumeister et al., 1990) and thus feel pressure to offer forgiveness even if not authentically felt, resulting in pseudo‐forgiveness, which appears conciliatory but can carry residual desire for vengeance (Quinney, Wenzel, Thai, et al., 2024; Takada & Ohbuchi, 2013). The present research set out to investigate whether empathy may reduce offenders' victimhood perceptions following victims' refusal to forgive. However, empathy may also have a dark side, whereby more empathetic people may have higher expectations of their empathy being reciprocated in social interactions (Bohns & Flynn, 2021) and thus hinder the reconciliation process.

### Forgiveness and non‐forgiveness

Researchers commonly conceptualize forgiveness as victims' prosocial change towards offenders involving an increase in benevolence and a decrease in revenge and avoidance motives (McCullough & Hoyt, 2002). Victims may express forgiveness to the offender (Wohl et al., 2006), possibly to regulate their unforgiving emotions (Bono et al., 2008) or for other expected benefits such as conflict avoidance or relationship repair. Victims may also communicate forgiveness because they understand it is what offenders may expect after they have apologized (Risen & Gilovich, 2007). However, sometimes victims may decide *not* to forgive offenders, even those who have apologized. Understanding forgiveness as a ‘gift’, victims have the right to offer or withhold it (Enright, 2019).

There are many reasons why victims may withhold forgiveness. Victims may perceive that offenders failed to provide a complete apology (Scher & Darley, 1997), appear to be trying to explain away their actions (Schumann, 2014) or to be apologizing only because they are expected to (Okimoto et al., 2015). Alternatively, victims may perceive the apology as coming too early because they still have unmet needs such as wanting to voice their concerns (Frantz & Bennigson, 2005) or to know the full truth (Quinney, Wenzel, & Woodyatt, 2024). Thus, victims may withhold forgiveness from an apologetic offender because they want *more* from the offender before they grant forgiveness. Of course, non‐forgiveness may also follow from the severity of the offence, repeated violations of trust, a want for revenge or other situational features (including dispositional factors) which mean that forgiveness may never be fully granted (Pearce et al., 2018). Nonetheless, whether victims express forgiveness or non‐forgiveness is their decision (Exline & Baumeister, 2000).

Yet, this decision is not without consequence. Non‐forgiveness sustained over time tends to make offenders defensive, which predictably has poor downstream consequences for victims, offenders and their relationships (Woodyatt & Wenzel, 2013). Victims may continue to feel weak/humiliated (Woodyatt et al., 2022), a sense of injustice (Foster & Rusbult, 1999) and still carry the unresolved needs or hurt that may have prompted their refusal to forgive. Offenders may lose motivation to engage in further reparative efforts and, so, may also miss opportunities to receive forgiveness in the future, and the ability to genuinely forgive themselves for their wrongdoing (Woodyatt & Wenzel, 2013), deal with their shame (vanOyen‐Witvliet et al., 2002) and satisfy their need for acceptance and restore their moral‐social identity (SimanTov‐Nachlieli & Shnabel, 2014). The relationship in which the transgression occurred also remains damaged, which can lead to conflict escalation, further wrongdoings and relationship dissolution (Feeney & Fitzgerald, 2019).

The present research investigates offenders' responses to receiving victims' non‐forgiveness after they have apologized. In general, people tend to react negatively to perceived rejection (Leary et al., 2006). However, apologetic offenders may feel particularly rejected because they may believe that they made the costly but correct decision to apologize and may see victims' refusal of forgiveness as disrespectful or an overreaction (Baumeister et al., 1990). At the intergroup level, research demonstrates that members of an offender group respond with increased anger and increased prejudice toward victim groups if those victim groups reject their apologies or offers of repair (Harth et al., 2011). At the interpersonal level, research demonstrates that offenders may perceive victims' non‐forgiveness as a new transgression for which they are the victim, and these victimhood perceptions can limit the prospect of reconciliation (Jennings et al., 2016; Thai et al., 2023). In other words, non‐forgiveness can have a role‐reversing effect whereby offenders believe that they too have been victimized at the hands of their victims.

### Offenders' victimhood perceptions

Empirical work indicates that offenders can see themselves as ‘victims’ when their apologies are rebuffed, which can limit offenders' efforts towards relational repair (Jennings et al., 2016). Specifically, researchers have proposed that non‐forgiveness makes offenders see themselves as victims, first because offenders interpret non‐forgiveness as a norm violation (Thai et al., 2023). Indeed, there can be normative expectations that offenders apologize after wrongdoing and that victims offer forgiveness in response to apologies (Fehr & Gelfand, 2012). Accordingly, apologetic offenders may believe that they have acted in line with what is expected of them and may see victims' refusal of forgiveness as having violated what was expected of them in return (Dhami, 2016). Hence, apologetic offenders may interpret victims' non‐forgiveness as violating a reciprocity norm that offenders may believe constitutes a separate transgression that makes them the victim.

The second pathway that might explain why non‐forgiveness elicits victimhood perceptions is through a perceived threat to the offender's status/power (Thai et al., 2023). By *threat to status/power*, we refer to a threat to *status*, including a diminished sense of perceived prestige, respect and esteem (Blader & Chen, 2012), but also a diminished sense of *power* or control over outcomes and resources (Karremans & Smith, 2010). Researchers propose that offenders first usurp power from victims by committing a transgression, but offenders may transfer power back to victims by apologizing (Okimoto et al., 2013). Yet, when offenders apologize first, this places them in a vulnerable position because they have transferred power to victims who are now in a position of power over them (Wenzel & Okimoto, 2010). Consequently, victims may use their position of power to forgive (and restore a power equilibrium; Shnabel et al., 2009) or instead assert themselves by refusing to forgive. If victims do not forgive, then apologetic offenders, who already feel vulnerable, may feel even further deprived of status/power—they are made to look foolish and/or feel weak, an experience that they may interpret as a new transgression that victimizes them.

Critically, this self‐perceived victimhood may distort offenders' identification as the offending party and inhibit them from engaging in further conciliatory action. In four empirical studies, Thai et al. (2023) evidenced this pattern, illustrating that non‐forgiveness can lead offenders to feel like victims—and through self‐perceived victimhood, to lower willingness to reconcile, and a greater likelihood to reoffend, avoid the victim and seek revenge against them.

### Do empathic offenders feel less like victims when forgiveness is withheld?

We theorized a priori that offenders' trait empathy might reduce their victimhood perceptions in response to non‐forgiveness. Empathy—the capacity to experience and understand another's cognitive and/or emotional states (Cuff et al., 2016)—can be effective in conflict resolution (Schumann & Dragotta, 2021; Zaki, 2014). Thai et al. (2023) considered whether a perspective‐taking prompt (vs. no perspective‐taking prompt) would moderate the effect of non‐forgiveness but found no effect. However, the task was only brief, and it is possible that brief perspective‐taking and/or state empathy prompts may be too weak to mitigate offenders' negative reaction to victims' non‐forgiveness (McAuliffe et al., 2020). In this study, we test the effect of empathy using measures of trait empathy.

In the current research, we sought to interrogate offenders' trait empathy (in particular, the facet of *empathic concern*) as a potential moderator of offenders' victimhood perceptions. Empathic concern is an other‐oriented emotional response towards unfortunate others, which includes feelings of concern, sympathy and compassion (Davis, 1983). Offenders higher in empathic concern may have a greater capacity to recognize or vicariously experience how their wrongdoing hurt the victim (Schumann & Dragotta, 2021) as well as a greater tendency to care about how the victim is feeling, which could increase their willingness to reconcile (Zaki, 2014). Thus, empathic offenders may be more understanding of the victims' non‐forgiveness rather than interpret non‐forgiveness as a norm violation, and a new transgression for which they are the victim. We specifically expected that empathic offenders may better recognize or appreciate that victims may have their reasons not to forgive them (e.g., still emotionally upset by the incident), and that granting forgiveness or not is their right, hence, empathic offenders may perceive that victims' non‐forgiveness does not constitute a norm violation. Indeed, people with greater empathy for the suffering of others may have a lower tendency to claim victimhood status across interpersonal situations and relationships (Gabay et al., 2020).

Although this appears to be a straightforward prediction, there is also a potential dark side to empathy. Some scholars have argued that empathy can produce harm (Bloom, 2017; Decety, 2021). Specifically, empathic concern can simultaneously increase recognition of other's emotions and increase negative moral evaluations (Batson, 2023). The dark side of empathic concern appears to emerge in antagonistic, threatening or competitive contexts. For example, empathic concern may fuel schadenfreude towards rival political group supporters who are injured during a political protest (Simas et al., 2020) and potentially mitigate justice concerns when interracial injustices have been committed (Johnson et al., 2024; Johnson & Lecci, 2020). Yet, these examples of the dark side of empathic concern are in an intergroup context rather than interpersonal conflict. In the context of interpersonal conflict, offenders' empathy appears to drive offenders towards reconciliation rather than deter them (Klimecki, 2019). Hence, a priori, we predicted that empathic concern would decrease offenders' self‐perceived victimhood after receiving non‐forgiveness.

Yet, if there is a dark side to offenders' empathic concern for resolving interpersonal conflict, then this may arise out of empathetic offenders' expectations for empathy. Previous research demonstrates that empathetic people have higher expectations that others are empathic, which may be constructive in social interactions as empathetic people believe that they can ask for help when they need it (Bohns & Flynn, 2021). However, expectations for empathy could be potentially destructive when the help, the empathic response, is withheld. Highly empathetic offenders who have apologized but receive non‐forgiveness in return may react even more negatively because they hold greater expectations of receiving an empathic response in return (e.g., forgiveness). That is, victims who refuse forgiveness may more strongly violate empathic offenders' expectations for empathy in return, leading highly empathic offenders to see themselves as even more like victims.

## THE PRESENT RESEARCH

The current research investigated whether offenders' trait empathic concern might help prevent offenders from seeing themselves as victims after non‐forgiveness, based on the reasoning that an empathic offender may have a greater capacity to recognize and understand that their transgression inflicted hurt on the victim. In Study 1, we pre‐registered our prediction that empathic concern would mitigate the effect of non‐forgiveness on victimhood perceptions by reducing norm violation perceptions. However, in later studies, we pre‐registered different predictions in light of the findings from Study 1.

### Transparency and openness

We pre‐registered all studies including study design, hypotheses, power analyses, inclusion/exclusion criteria and pre‐planned primary analyses.^1^ The pre‐registrations for these studies can be accessed via the Open Science Framework link: https://osf.io/tvmk4/?view_only=94f8582ea33b4e9b868dfcc9c4eb5bf3 (Study 1), https://osf.io/8f6jn/?view_only=f20dd2524c944717a154383177314a03 (Study 2), https://osf.io/f2vew/?view_only=e9e82a83aee44580a9cfeaa1f4815766 (Study 3). The data, analysis code, research materials, Data S1 and further analyses for the present research are available at [https://osf.io/c7kmx/?view_only=f2db7a24bcb74506a4a055a01f0b77af].

## STUDY 1

In Study 1, we investigated offenders' victimhood perceptions following non‐forgiveness. We tested whether the pathway from non‐forgiveness (vs. forgiveness) to norm violation perceptions would be weaker for offenders high (vs. low) in empathic concern, which would in turn translate into decreased victimhood perceptions. We explored whether empathic concern would have a similar mitigating effect but via reducing threat to status/power.

### Method

#### Participants

We conducted a Monte Carlo simulation power analysis for a moderated mediation in Mplus v8.8 (Muthén & Muthén, 2017). Based on effect sizes found by Thai et al. (2023, Study 1, which used a similar methodology) and assuming a standardized regression coefficient of 0.22 for the moderation effects of empathy, this determined that we would need 300 participants to have power >0.80 to detect the moderations and implied conditional indirect effects on victimhood perceptions. We recruited residents of the United States through Amazon's Mechanical Turk, via the recruitment platform, CloudResearch (Litman et al., 2017).

We screened participants from the study if they indicated that they could not recall separate instances of receiving forgiveness and non‐forgiveness after apologizing for a wrongdoing. This allowed us to randomly allocate participants to recall conditions given that participants must have experienced both forgiveness and non‐forgiveness. We excluded data from participants who failed to complete all dependent variables or follow the relevant condition instructions of describing a wrongdoing where they were forgiven by the victim in the forgiveness condition (and vice versa). The final sample after screening and exclusions was 297 participants (150 men, 142 women, 1 non‐binary; *M*_age_ = 39.6). Of these, 76.5% identified as White, 10.2% African American, 6.8% Asian, 4.4% Hispanic or Latino and 2.0% Multiracial.

#### Design and procedure

This study was a two‐cell design (victim response to apology: forgiveness; non‐forgiveness). Participants first completed the empathy trait measure before we randomly allocated them to recall an incident when they committed a wrongdoing and were either forgiven or not forgiven by the victim. The same instructions were used from Thai et al. (2023): *Please take a moment to recall an incident where you committed a wrongdoing against someone else, you apologized wholeheartedly to the other person, and they forgave you* (forgiveness condition) *and they did not forgive you* (non‐forgiveness condition).

We asked participants to describe their transgression, indicate what their relationship status was with the other person (29.9% significant other, 29.3% close friend, 18.4% family member, 10.9% work colleague, 5.8% acquaintance, 3.4% stranger, 2.4% other) and categorize their wrongdoing (31.6% betrayal of trust, other 31.0%, 17.2% insult, 10.4% infidelity, 6.8% betrayal of confidence, 2.7% physical abuse). Participants' other responses tended to indicate the wrongdoing was negligent (e.g., they caused a road accident). Participants then completed the dependent variables and demographic questions.

##### Measures

We used 7‐point Likert scales to measure each item, unless otherwise indicated (1 = strongly disagree, 7 = strongly agree). Each scale presented its items in a random order.

###### Trait empathy

We used the *Interpersonal Reactivity Index* (IRI; Davis, 1983) to capture participants' trait empathy. The IRI is a self‐report, 28‐item scale that divides into four 7‐item subscales including: empathic concern (*α* = .89), personal distress (*α* = .87), fantasy (*α* = .85) and perspective taking (*α* = .85). Participants responded to each item on a scale of 1 = ‘Does not describe me well’ to 5 = ‘Describes me very well’.

###### Victimhood perceptions

One item from Thai et al. (2023) assessed the extent to which participants felt like the victim after receiving the victim's response to their apology: ‘To what extent did you feel like a victim in this situation?’ (1 = not at all, 7 = very much).

###### Norm violation perceptions

The 5‐item scale from Thai et al. (2023) assessed whether participants believed how the victim responded to their apology was a violation of the norms for expected behaviour (e.g., ‘This person has not responded how a person would be expected to respond under the circumstances’; *α* = .91).

###### Threat to status/power

The 6‐item scale from Thai et al. (2023) assessed participants' perceived threat to their status/power as a result of the victims' response to their apology (e.g., ‘This person's response makes me feel like a fool’, *α* = .77).

### Results

We conducted independent *t*‐tests to analyse the effect of non‐forgiveness (vs. forgiveness) on our main dependent variables. Table 1 presents the descriptive statistics, *t*‐tests and effect sizes. Participants (*N* = 147) in the non‐forgiveness condition reported greater norm violation perceptions, greater threat to status/power, and greater victimhood perceptions, than participants in the forgiveness condition (*N* = 150). Table 2 presents the bivariate correlations between empathic concern and these dependent variables.

**TABLE 1:** Descriptive and inferential statistics for main variables (Study 1).

Variable	Victim response to apology		*t* (295)	*d* [95% CI]
	Forgiveness *M* (SD)	Non‐forgiveness *M* (SD)		
1. Victimhood perceptions	1.64 (1.27)	2.69 (1.87)	−5.69	0.66 [0.43, 0.89]
2. Norm violation perceptions	2.02 (0.90)	3.89 (1.55)	−12.8	1.48 [1.22, 1.74]
3. Threat to status/power	3.19 (0.84)	4.96 (0.99)	−16.7	1.94 [1.66, 2.21]

*Note*: All *p* values are significant at < .001.

**TABLE 2:** Bivariate correlations for outcome and trait empathy variables (Study 1).

Variable	Correlations			
	1	2	3	4
1. Empathic concern	–	−.18**	−.16**	−.06
2. Victimhood perceptions		–	.61***	.31***
3. Norm violation perceptions			–	.49***
4. Threat to status/power				–

*Note*: **p < .01, ***p < .001.

#### Moderation by empathic concern

We used Process Model 7 (Hayes, 2022) to test our moderated mediation hypothesis. Conditional effects were calculated at –1SD and +1SD to represent low and high levels of the moderator, respectively. Table 3 presents the results.

**TABLE 3:** Moderated mediation of empathic concern on the effect of non‐forgiveness on victimhood perceptions via norm violation perceptions (Study 1).

Variable	*B*	SE *B*	*p*	CI_95%_
Norm violation perceptions		*R* = .62, *R* ^2^ = .38, *p* < .001		
Constant	−1.74	0.14	<.001	[−2.02, −1.45]
Non‐forgiveness	1.17	0.09	<.001	[0.99, 1.35]
Empathic concern	−0.51	0.15	<.001	[−0.80, −0.22]
Non‐forgiveness × Empathic concern	0.26	0.09	.005	[0.08, 0.44]
High empathic concern (simple slope)	1.43	0.13	<.001	[1.18, 1.69]
Low empathic concern (simple slope)	0.91	0.13	<.001	[0.65, 1.16]
Victimhood perceptions		*R* = .61, *R* ^2^ = .38, *p* < .001		
Constant	0.23	0.18	.193	[−0.12,0.58]
Non‐forgiveness	−0.15	0.11	.177	[−0.38, 0.07]
Norm violation perceptions	0.66	0.06	<.001	[0.54, 0.77]
Index of moderated mediation	0.17	0.07	–	[0.04, 0.32]
High empathic concern (conditional indirect effect)	0.94	0.13	–	[0.71, 1.21]
Low empathic concern (conditional indirect effect)	0.60	0.11	–	[0.39, 0.83]

Non‐forgiveness (vs. forgiveness) was significantly positively associated with norm violation perceptions, and empathic concern was significantly negatively associated with norm violation perceptions. Empathic concern significantly moderated the effect of non‐forgiveness (vs. forgiveness) on norm violation perceptions. However, the moderating effect of empathic concern was in the opposite direction to our predictions, as it *strengthened* the effect of non‐forgiveness on norm violation perceptions. Indeed, tests of simple slopes found a stronger positive association between non‐forgiveness (vs. forgiveness) and norm violation perceptions for those high in empathic concern than for those low in empathic concern. Furthermore, the index of moderated mediation indicated a significant moderating effect of empathic concern on the effect of non‐forgiveness (vs. forgiveness) on victimhood perceptions via norm violation perceptions. Again, contrary to expectations, the conditional indirect effect was more strongly positive for those high in empathic concern and weaker but still positive for those low in empathic concern.

##### Exploratory moderated mediation analysis

We present these results in the Data S1. We tested whether empathic concern may also moderate the effect of non‐forgiveness (vs. forgiveness) on victimhood perceptions via threat to status/power. Indeed, like norm violation perceptions, empathic concern significantly strengthened the effect of non‐forgiveness on threat to status/power, but the index of moderated mediation was non‐significant.

### Discussion

In this study, contrary to our predictions, we found that empathic concern *increased* the effect of non‐forgiveness on norm violation perceptions, which in turn was associated with greater victimhood perceptions. We refrain here from interpreting this contradictory effect as we remained skeptical about this finding. Thus, we conducted Study 2 to test whether this unexpected effect of empathic concern would replicate.

## STUDY 2

Study 2 was a replication attempt using a UK sample instead of a US sample. We decided to examine whether the unexpected findings would replicate in a different population to increase the generalisability of the findings. Yet, we retained population features such as having English as the primary language to avoid introducing significant variability due to language and/or significant cultural differences. We pre‐registered the same empathic concern moderation hypotheses as Study 1, but we also pre‐registered predictions in line with the previous findings of Study 1. Thus, we made a competing prediction that the relationship between non‐forgiveness vs. forgiveness and norm violation perceptions will be stronger, the higher participants are in empathic concern. We also explored again the moderating effect of empathic concern on threat to status/power.

### Method

#### Participants

We relied on the previous power analysis conducted for Study 1 and collected data from 300 participants. We recruited residents of the United Kingdom via the recruitment platform, Prolific. A total of 300 participants provided complete data. We excluded data from 4 participants for describing an instance when they were forgiven by the victim in the non‐forgiveness condition or vice versa. Thus, we retained data from 296 participants for analysis (238 women, 58 men; *M*_age_ = 36.8). Of these, 85.5% identified as White/White British, 9.5% Asian/Asian British, 2.0% Black/African/Caribbean British, 2.0% Multiracial, 0.3% Hispanic, 0.3% Italian, and 0.3% left it unspecified.

#### Design and procedure

Participants followed the same design and procedure as Study 1. Again, participants described what their relationship status was with the other person (37.8% close friend, 16.9% significant other, 16.2% family member, 15.5% work colleague, 6.8% acquaintance, 4.1% other, 2.7% stranger) and categorized their wrongdoing (35.1% other, 27.0% betrayal of trust, 21.6% insult, 11.1% betrayal of confidence, 4.7% infidelity, 0.3% physical abuse).

#### Measures

We used the same scales from Study 1 to measure empathic concern (*α* = .84), victimhood perceptions (single item), norm violation perceptions (*α* = .92) and threat to status/power (*α* = .77).

### Results

We conducted independent *t*‐tests to analyse the effect of non‐forgiveness (vs. forgiveness) on all dependent variables. Table 4 presents the descriptive statistics, *t*‐tests and effect sizes. Replicating the findings of Study 1, participants (*N* = 150) in the non‐forgiveness condition reported greater norm violation perceptions, greater threat to status/power and greater victimhood perceptions than participants in the forgiveness condition (*N* = 146). Table 5 presents the correlations between empathic concern and these dependent variables.

**TABLE 4:** Descriptive and inferential statistics for main variables (Study 2).

Variable	Victim response to apology		*t* (294)	*d* [95% CI]
	Forgiveness *M* (SD)	Non‐forgiveness *M* (*SD*)		
1. Victimhood Perceptions	2.03 (1.54)	3.43 (2.09)	−6.57	0.76 [0.53, 1.00]
2. Norm Violation Perceptions	2.39 (1.01)	4.06 (1.52)	−11.1	1.29 [1.04, 1.54]
3. Threat to Status/Power	3.60 (0.89)	5.05 (1.08)	−12.6	1.47 [1.21, 1.73]

**TABLE 5:** Bivariate correlations for outcome and trait empathy variables (Study 2).

Variable	Correlations			
	1	2	3	4
1. Empathic concern	–	.02	.01	.10
2. Victimhood perceptions		–	.59***	.52***
3. Norm violation perceptions			–	.50***
4. Threat to status/power				–

*Note*: *p < .05, **p < .01, ***p < .001.

#### Test for moderation of empathic concern on norm violation perceptions

We used Process Model 7 (Hayes, 2022) to test our moderated mediation hypothesis. Table 6 presents the results. However, contrary to Study 1, empathic concern did not significantly moderate the effect of non‐forgiveness (vs. forgiveness) on norm violation perceptions (*b* = 0.11, SE = 0.10, *p* = .282).

**TABLE 6:** Moderated mediation of empathic concern on the effect of non‐forgiveness on victimhood perceptions via norm violation perceptions (Study 2).

Variable	*B*	SE *B*	*p*	CI95%
Norm violation perceptions		*R* = .55, *R* ^2^ = .30, *p* < .001		
Constant	−1.65	0.16	<.001	[−1.96, −1.35]
Non‐forgiveness	1.09	0.10	<.001	[0.90, 1.29]
Empathic concern	−0.21	0.16	.200	[−0.52, 0.11]
Non‐forgiveness × Empathic concern	0.11	0.10	.282	[−0.09, 0.30]
Victimhood perceptions		*R* = .59, *R* ^2^ = .35, *p* < .001		
Constant	−0.16	0.18	.350	[−0.51, 0.18]
Non‐forgiveness	0.11	0.11	.332	[−0.11, 0.33]
Norm violation perceptions	0.56	0.06	<.001	[0.45, 0.67]
Index of moderated mediation	0.06	0.05	–	[−0.05, 0.17]

##### Exploratory moderated mediation analysis

In additional analyses, empathic concern significantly moderated the effect of non‐forgiveness (vs. forgiveness) on threat to status/power (see Table 7). We found a significantly stronger conditional indirect effect of non‐forgiveness on victimhood perceptions via increased threat to status/power for participants high (vs. low) in empathic concern.

**TABLE 7:** Exploratory moderated mediation analysis of empathic concern on the effect of non‐forgiveness on victimhood perceptions via threat to status/power (Study 2).

Variable	*B*	SE *B*	*p*	CI_95%_
Threat to status/power		*R* = .62, *R* ^2^ = .38, *p* < .001		
Constant	−1.79	0.15	<.001	[−2.08, −1.51]
Non‐forgiveness	1.18	0.09	<.001	[1.00, 1.36]
Empathic concern	−0.48	0.15	<.001	[−0.78, −0.19]
Non‐forgiveness × Empathic concern	0.34	0.09	<.001	[0.16, 0.52]
High empathic concern (simple slope)	1.52	0.13	<.001	[1.26, 1.78]
Low empathic concern (simple slope)	0.84	.13	<.001	[0.58, 1.09]
Victimhood perceptions		*R* = .53, *R* ^2^ = .28, *p* < .001		
Constant	−0.22	0.18	.259	[−0.60, 0.16]
Non‐forgiveness	0.14	0.12	.243	[−0.10, 0.39]
Threat to status/power	0.48	0.06	<.001	[0.36, 0.60]
Index of moderated mediation	0.16	0.05	–	[0.07, 0.28]
High empathic concern (conditional indirect effect)	0.73	0.12	–	[0.51, 0.96]
Low empathic concern (conditional indirect effect)	0.40	0.09	–	[0.24, 0.59]

### Discussion

Unlike Study 1, we found no statistically significant moderating effect of empathic concern on norm violation perceptions, perhaps due to a smaller sized interaction effect. Yet, an exploratory analysis revealed that empathic concern significantly enhanced the effect of non‐forgiveness on threat to status/power. That is, people high in empathic concern (vs. low) experienced a more exacerbated threat to status/power in response to non‐forgiveness, which in turn was associated with stronger victimhood perceptions. Hence, the evidence collected so far did not support our a priori assumption that empathic concern would mitigate the effect of non‐forgiveness on offenders' victimhood perceptions. On the contrary, the evidence suggested that offenders who are higher in empathic concern tend to be particularly aggrieved by victims' non‐forgiveness. Thus, we conducted Study 3 to understand why this may be the case.^2^

## STUDY 3

Why would trait empathy increase the effect of non‐forgiveness on apologetic offenders' feelings of victimhood? One possibility is that empathic individuals also expect greater empathy from others (Bohns & Flynn, 2021). First, it is possible that empathic people hold empathy as a general norm that they expect others to live and act by, too (normative expectation for empathy). Second, empathic people may perceive empathy as a commodity to be traded, and given that the offenders apologized to the victim, they may expect reciprocity from victims (reciprocity expectation for empathy). Alternatively, empathic people may regard the value of empathy as unconditional and, even though they harmed the victim initially, they expect it to be extended to them (unconditional expectation for empathy).

We therefore predicted an equivalent interaction between non‐forgiveness and empathy expectations (expectations for empathy will increase the effect of non‐forgiveness on norm violation perceptions and threat to status/power), which would fully mediate the moderating effect of empathic concern (i.e., empathic concern would no longer significantly moderate the effect of non‐forgiveness on norm violation perceptions, and threat to status/power). This hypothesis is equivalent to a mediated moderation hypothesis and highlights our theorizing that it is the expectations for empathy that derive from empathic concern that are the potentially problematic aspect, rather than empathic concern per se (see Muller et al., 2005; Wenzel et al., 2012).

### Method

#### Participants

We conducted a Monte Carlo simulation power analysis for moderation in Mplus v8.8 (Muthén & Muthén, 2017). The power analysis determined that we would need 400 participants to have power >0.80 to detect a standardized interaction coefficient of 0.20. We requested data from a total of 420 participants to account for any power loss due to exclusions. We recruited residents of the United Kingdom via the recruitment platform, Prolific. However, we excluded data from 13 participants for either failing more than one attention check or describing a time when they were forgiven by the victim in the non‐forgiveness condition or vice versa. Thus, data from 407 participants were eligible for analysis (272 women; 134 men; 1 non‐binary; *M*_age_ = 39.8). Of these, 87.5% identified as White/White British, 5.4% Asian/Asian British, 2.9% Black/African/Caribbean British, 2.9% Multiracial, 0.2% Hispanic, 0.2% Polish and 0.9% left it unspecified.

#### Design and procedure

Participants followed the same design and procedure as Studies 1 and 2. First, participants indicated their relationship status with the victim (36.9% close friend, 22.6% significant other, 15.2% family member, 14.3% work colleague, 5.4% acquaintance, 2.9% other, 2.7% stranger) and categorized their wrongdoing (34.6% other, 26.3% betrayal of trust, 20.6% insult, 8.4% infidelity, 8.1% betrayal of confidence, 2.0% physical abuse).

#### Measures

We used the same scale to measure empathic concern (*α* = .84), victimhood perceptions (single item), norm violation perceptions (*α* = .90) and threat to status/power (*α* = .75). We included three additional items to capture participants' perceptions of their transgression. These included severity (‘How serious do you believe your wrongdoing was?’), intentionality (‘How intentional was your wrongdoing?’) and forgivability (‘How much do you believe you deserve to be forgiven for your wrongdoing?’). Participants responded to these items on a scale of 1 = not at all to 7 = very much (Thai et al., 2023).

We developed three sets of empathy expectations scales with four items each that had different foci (i.e., normative expectation, reciprocity expectation, and unconditional expectation). We pre‐registered our decision to conduct a Principal Component Analysis and let the data guide us as to whether we construct a single empathy expectations scale or separate scales. A principal components analysis yielded three components from the initial extraction with eigenvalues greater than one, and as indicated by the scree plot. We conducted a Varimax rotation with Kaiser Normalization and each item loaded strongly (>0.85) onto their relevant empathy expectation scale. We thus constructed three separate empathy expectations scales.

##### Normative expectation for empathy

Four items assessed participants' belief that people should be empathetic toward others in distress (e.g., ‘People should be empathetic when others are hurting’; *α* = .94).

##### Reciprocity expectation for empathy

Four items assessed participants' belief that people should reciprocate empathy with empathy (e.g., ‘If a person has been empathetic toward others who were hurting, those others should be empathetic to that person in return when the person is hurting’; *α* = .97).

##### Unconditional expectation for empathy

Four items assessed participants' belief that people should be empathetic towards others even if those others have not demonstrated empathy (e.g., ‘If a person has not been empathetic toward others who were hurting, those others should still be empathetic to that person when the person is hurting’; *α* = .98).

### Results

We conducted independent *t*‐tests to analyse the effect of non‐forgiveness (vs. forgiveness) on all dependent variables. Table 8 presents the descriptive statistics, *t*‐tests and effect sizes. Replicating the findings of Studies 1 and 2, participants (*N* = 203) in the non‐forgiveness condition reported greater norm violation perceptions, greater threat to status/power and greater victimhood perceptions, than participants in the forgiveness condition (*N* = 204). Participants in the forgiveness condition reported significantly greater perceived severity of their transgression than participants in the non‐forgiveness condition, but there were no significant between group differences on perceived intentionality or perceived forgivability.^3^ Table 9 presents the bivariate correlations between the main dependent variables and trait measures.

**TABLE 8:** Descriptive and inferential statistics for main variables in Study 3.

Variable	Victim response to apology		*t* (405)	*p*	*d* [95% CI]
	Forgiveness *M* (SD)	Non‐forgiveness *M* (SD)			
1. Victimhood perceptions	1.73 (1.25)	3.14 (1.90)	−8.89	<.001	0.88 [0.68, 1.08]
2. Norm violation perceptions	2.35 (0.88)	4.13 (1.37)	−15.7	<.001	1.55 [1.33, 1.78]
3. Threat to status/power	3.48 (0.81)	4.96 (0.94)	−17.1	<.001	1.69 [1.46, 1.92]
4. Perceived severity	4.24 (1.65)	3.78 (1.79)	2.68	.008	0.27 [0.70, 0.46]
5. Perceived intentionality	2.82 (1.88)	2.71 (1.90)	0.58	.560	0.06 [−0.14, 0.25]
6. Perceived forgivability	5.36 (1.36)	5.11 (1.56)	0.09	.086	0.17 [−0.02, 0.37]

**TABLE 9:** Bivariate correlations for outcome and trait empathy variables in Study 3.

Variable	Correlations						
	1	2	3	4	5	6	7
1. Empathic concern	–	.02	.05	.08	.64***	.31***	.33***
2. Victimhood perceptions		–	.63***	.45***	.01	.05	.06
3. Norm violation perceptions			–	.54***	.02	.02	.01
4. Threat to status/power				–	.08	.06	.14**
5. Normative expectation for empathy					–	.47***	.44***
6. Reciprocity expectation for empathy						–	.26***
7. Unconditional expectation for empathy				‐			–

*Note*: **p < .01, ***p < .001.

#### Moderation by empathic concern

We used Process Model 7 (Hayes, 2022) to test our two moderated mediation hypotheses. Table 10 presents the results for the model with norm violation perceptions and threat to status/power as the mediators (tested in the same analysis). Non‐forgiveness (vs. forgiveness) significantly positively predicted norm violation perceptions and threat to status/power. Empathic concern was significantly negatively related to norm violation perceptions and threat to status/power; and, as predicted, empathic concern significantly moderated the effect of non‐forgiveness (vs. forgiveness) on norm violation perceptions and threat to status/power. The tests of simple slopes suggested that there was a stronger positive association between non‐forgiveness (vs. forgiveness) and norm violation perceptions and threat to status/power for those high in empathic concern than low in empathic concern. Both indices of moderated mediation were also significant. The pattern of conditional indirect effects indicated there were stronger positive indirect effects of non‐forgiveness to victimhood perceptions via norm violation perceptions and threat to status/power for those high in empathic concern than those low in empathic concern.

**TABLE 10:** Moderated mediation of empathic concern on the effect of non‐forgiveness on victimhood perceptions via norm violation perceptions and threat to status/power (Study 3).

Variable	*B*	SE *B*	*p*	CI_95%_
Norm violation perceptions		*R* = .63, *R* ^2^ = .40, *p* < .001		
Constant	0.01	0.04	.977	[−0.07, 0.08]
Non‐forgiveness	0.61	0.04	<.001	[0.54, 0.69]
Empathic concern	0.05	0.04	.203	[−0.03, 0.13]
Non‐forgiveness × Empathic concern	0.13	0.04	<.001	[0.06, 0.21]
High empathic concern (simple slope)	0.75	0.05	<.001	[0.64, 0.85]
Low empathic concern (simple slope)	0.48	0.05	<.001	[0.37, 0.59]
Threat to status/power		*R* = .66, *R* ^2^ = .43, *p* < .001		
Constant	0.01	0.04	.974	[−0.07, 0.07]
Non‐forgiveness	0.65	0.04	<.001	[0.57, 0.72]
Empathic concern	0.07	0.04	.047	[0.01, 0.15]
Non‐forgiveness × Empathic concern	0.11	0.04	.002	[0.04, 0.19]
High empathic concern (simple slope)	0.76	0.05	<.001	[0.66, 0.86]
Low empathic concern (simple slope)	0.53	0.05	<.001	[0.43, 0.63]
Victimhood perceptions		*R* = .65, *R* ^2^ = .42, *p* < .001		
Constant	−0.01	0.04	.996	[−0.08, 0.07]
Non‐forgiveness	−0.07	.05	.187	[−0.18, 0.04]
Norm violation perceptions	0.58	0.05	<.001	[0.48, 0.67]
Index of moderated mediation	0.08	0.02	–	[0.03, 0.12]
High empathic concern (conditional indirect effect)	0.43	0.05	–	[0.34, 0.53]
Low empathic concern (conditional indirect effect)	0.28	0.04	–	[0.20, 0.36]
Threat to status/power	0.19	0.05	<.001	[0.09, 0.29]
Index of moderated mediation	0.02	0.01	–	[0.01, 0.04]
High empathic concern (conditional indirect effect)	0.14	0.05	–	[0.06, 0.23]
Low empathic concern (conditional indirect effect)	0.10	0.03	–	[0.04, 0.16]

#### Mediated moderation by expectations for empathy

We conducted hierarchical regression analyses to test our hypotheses that it is expectations for empathy that derive from empathic concern which cause the non‐forgiveness enhancing effects. In Step 1, we included non‐forgiveness (vs. forgiveness), empathic concern and the interaction term between non‐forgiveness and empathic concern, which were significantly positively related to norm violation perceptions and threat to status/power (consistent with the previous moderated mediation analyses). In Step 2, we added each expectation for empathy scale as predictors, and three interaction terms between these expectations for empathy and non‐forgiveness. We investigated our hypotheses that the interactions between expectations for empathy and non‐forgiveness would mirror the interactions between empathic concern and non‐forgiveness on norm violation perceptions and threat to status/power. Further, we expected that the empathic concern and non‐forgiveness interaction effect would no longer be statistically significant after controlling for the interactions between expectations for empathy and non‐forgiveness.

We present the results for the hierarchical regressions conducted for norm violation perceptions and threat to status/power in Tables 11 and 12, respectively. In Step 2, the interaction term between reciprocity expectation for empathy and non‐forgiveness emerged as the only interaction effect that significantly positively predicted norm violation perceptions (*β* = 0.15, *p* < .001) and threat to status/power (*β* = 0.09, *p* = .036). We calculated conditional effects at –1SD and +1SD to represent low and high levels of reciprocity expectation for empathy. For those high vs. low in reciprocity expectation for empathy, we found a *stronger* positive association between non‐forgiveness (vs. forgiveness) and norm violation perceptions (*β* = 1.15, *p* < .001 vs. *β* = 0.64, *p* < .001) and threat to status/power (*β* = 0.90, *p* < .001 vs. *β* = 0.58, *p* < .001). Further, as predicted, the interaction term between non‐forgiveness and empathic concern no longer significantly predicted norm violation perceptions (*β* = 0.08, *p* = .105) and threat to status/power (*β* = 0.02, *p* = .623). This implies that the moderating effect of the reciprocity expectation for empathy accounts for the moderating effect of empathic concern on norm violation perceptions and threat to status/power.

**TABLE 11:** Standardized coefficients from hierarchical regression conducted on norm violation perceptions in Study 3.

	Step 1	Step 2
Norm violation perceptions		
Non‐forgiveness	0.61***	0.62***
Empathic concern	0.05	0.06
Non‐Forgiveness × Empathic concern	0.13***	0.08
Normative expect. emp.	–	−0.03
Reciprocity expect. emp.	–	0.02
Unconditional expect. emp.	–	−0.03
Non‐forgiveness × Normative expect. emp.	–	−0.01
Non‐forgiveness × Reciprocity expect. emp.	–	0.15***
Non‐forgiveness × Unconditional expect. emp.	–	0.03

*Note*: Normative expect. emp., normative expectation for empathy; Reciprocity Expect. Emp., reciprocity expectation for empathy, Unconditional Expect. Emp., unconditional expectation for empathy. ***p < .001.

**TABLE 12:** Standardized coefficients from hierarchical regression conducted on threat to status/power in Study 3.

	Step 1	Step 2
Threat to Status/Power		
Non‐forgiveness	0.65***	0.64***
Empathic concern	0.07*	0.03
Non‐forgiveness × Empathic concern	0.12**	0.02
Normative expect. emp.	–	−0.02
Reciprocity expect. emp.	–	0.04
Unconditional expect. emp.	–	0.10*
Non‐forgiveness × Normative expect. emp.	–	0.07
Non‐forgiveness × Reciprocity expect. emp.	–	0.09*
Non‐forgiveness × Unconditional expect. emp.	–	0.04

*Note*: Normative expect. emp., normative expectation for empathy; Reciprocity expect. emp., reciprocity expectation for empathy; unconditional expect. emp., unconditional expectation for empathy. *p < .05, ***p < .001.

### Discussion

In Study 3, we investigated why offenders higher in empathic concern might feel even more like victims in response to non‐forgiveness. We tested whether this effect might be explained by empathic concern leading to expectations for empathy. We therefore examined whether the moderating effect of empathic concern would be mirrored by, and fully mediated by, an equivalent interaction between non‐forgiveness and expectations for empathy. The findings supported these predictions. In particular, a *reciprocity* expectation for empathy appeared to account for the moderating effect of empathic concern. This suggests that people with higher trait empathic concern may expect empathy to be reciprocated with empathy by others. In cases of offending, an empathic offender apologizing for their wrongdoing might expect forgiveness in return. Yet, unforgiving victims violate this reciprocity expectation, leading empathic offenders to feel particularly victimized.

## GENERAL DISCUSSION

Apologetic offenders may believe that they have shown appropriate contrition for their wrongdoing and may expect victims to offer forgiveness in return. However, victims can refuse to forgive apologetic offenders, which offenders perceive as a norm violation, a threat to their status/power, and as a transgression for which they are the victim (Thai et al., 2023). In the present research, in stark contrast to our a priori theorizing, we found that offenders' trait empathic concern may enhance victimhood perceptions.

### The dark side of empathic concern for offenders' engagement in reconciliation

The present research illustrates that offenders' empathic concern may frustrate rather than facilitate reconciliation. Indeed, offenders higher in empathic concern viewed victims' non‐forgiveness as a greater norm violation (n.s. in Study 2) and a greater threat to their status/power. Consequently, empathic offenders felt more like victims via these perceptions. We found an explanation for this effect in Study 3. Empathic concern was positively associated with reciprocity expectations of empathy, which fully mediated and so accounted for the interaction of non‐forgiveness and empathic concern. Thus, empathic concern can imply expectations that others be empathic in return. Offenders higher in empathic concern may view victims' non‐forgiveness as a stronger violation of empathy reciprocity norms, leading offenders higher in empathic concern to feel particularly victimized.

Our findings that trait empathic concern exacerbated offenders' negative reaction to victims' non‐forgiveness dovetails with other criticisms of empathy (Bloom, 2017) and illustrates another case when empathic concern is implicated in enhancing interpersonal conflict (i.e., an ‘interpersonal liability’, Batson, 2023; see also Hu et al., 2020). The present research findings provide a contrast to restorative models of reconciliation that highlight the positive function of offenders' empathy (Wallis, 2014).

Indeed, the present research may highlight the paradoxical nature of offenders' empathy in victim–offender reconciliation. On the one hand, offenders' empathy might be critical in the initial stages following wrongdoing for offenders to recognize the effects on victims and engage in conflict resolution (Zaki & Cikara, 2015). Here, offenders' trait empathy might be an important initiator for relationship/moral repair especially for driving offenders to apologize (Howell et al., 2012; Leunissen et al., 2017). On the other hand, offenders' empathy may make them more likely to disengage from repair if their apologies are not met with forgiveness. Paradoxically, empathy may be critical for offenders to engage in repair, but empathy may also drive offenders to disengage from repair, if their initial efforts are unsuccessful.

The present research speaks to a concerning feature of trait empathic concern. Namely, people high in empathic concern may have greater expectations that others be empathic—an empathy quid pro quo—and react particularly negatively when others violate these expectations. Previous research has examined the positive aspects of expectations that derive from empathic concern. For example, people higher in empathic concern (vs. lower) may have greater expectations that others will help them, which might increase the likelihood of asking others for help (Bohns & Flynn, 2021). Our research suggests that there may be a dark side to expectations for empathy, as people higher in empathic concern may react particularly negatively towards others if others' behavior does not meet their expectations (e.g., if others refuse to help). The results of Study 3 suggest that the reciprocity expectation for empathy is the critical empathy expectation that explains the moderating effect of empathic concern on non‐forgiveness. Theoretically, the finding that reciprocity expectations for empathy drive the moderation of non‐forgiveness effects on norm violation perceptions is consistent with how offenders view victims' non‐forgiveness as violating an apology‐forgiveness reciprocity norm (Thai et al., 2023). More (vs. less) empathic offenders may more strongly expect that their apology be reciprocated with forgiveness and therefore experience non‐forgiveness as a greater norm violation. The finding that reciprocity expectations for empathy drive the moderation of non‐forgiveness effects on threat to status/power could reflect empathic offenders believing that, because they have apologized and showed respect to victims, they expect that victims should also treat them with respect by accepting their apology and/or granting forgiveness. Again, because non‐forgiveness violates this reciprocity expectation for empathy, empathic offenders may feel more disrespected and, therefore, particularly victimized.

### Limitations and future directions

The present research has limitations which should be considered when interpreting the present findings. Methodologically, we asked offenders to recall transgression scenarios that fit the experimental conditions. It is possible that offenders may have recalled qualitatively different transgressions depending on the experimental condition. For example, in Study 3, participants in the forgiveness condition reported greater perceived severity of their transgression than participants in the non‐forgiveness condition. This finding could reflect that offenders selectively recalled more serious situations when asked about incidents for which they were forgiven compared to when they were not forgiven. Another possibility is that the current standing of the relationship could affect how offenders recall their transgression. Future research could utilize experimental designs such as scenario‐based vignettes to manipulate forgiveness/non‐forgiveness and overcome the potential issues of selective recall. Alternatively, future research could capture within‐person change in attributions by employing prospective‐longitudinal methodology to capture transgressions in real time and the changing dynamics that unfold between victims and offenders over time (see Wenzel et al., 2021).

An additional limitation of the present research is that the studies used a measurement‐of‐mediator design (Pirlott & MacKinnon, 2016). We provided evidence that our non‐forgiveness manipulation directly affected all outcomes of interest (i.e., norm violation perceptions, threat to status/power and victimhood perceptions), and that empathic concern strengthened the relationships between non‐forgiveness and norm violation perceptions and non‐forgiveness and threat to status/power. However, the mediation component of the moderated mediation analyses only provided evidence of a correlational relationship between the presumed mediating variables (norm violation perceptions and threat to status/power) and outcome variable (victimhood perceptions). We note that the presumed mediator–outcome relationships are grounded in theory that links norm violation perceptions and threat to status/power to victimization (e.g., Shnabel et al., 2009), and previous research has also evidenced these mediator–outcome relationships (Thai et al., 2023). Nevertheless, future research should seek to substantiate these links by going beyond a measurement‐of‐mediator design to an experimental‐causal‐chain design (Spencer et al., 2005).

We measured trait empathy using the Interpersonal Reactivity Index (Davis, 1983) which is the most cited empathy scale, but it has limitations. One issue is that participants self‐report on their level of trait empathy, which could elicit self‐enhancement (Paulhus & Vazire, 2007). Thus, our findings could be limited to self‐perceived empathy, and we may doubt the accuracy of self‐reported empathy (cf. Depow & Inzlicht, 2025). A related concern is that we did not measure trait empathy using other validated measures to conceptually replicate our findings. Future research may further demonstrate the veracity of our findings by varying the operationalization of empathy.

A pertinent future direction is further to investigate the reciprocity expectation for empathy that derives from trait empathy. In particular, future research could utilize interventions to target these expectations for empathy which may be hijacking or perverting the prosocial elements of empathic concern. One approach could be to de‐emphasize the reciprocal expectation for empathy, perhaps by framing victims' forgiveness as a gift, or emphasizing the normative nature of non‐forgiveness. Another consideration could be whether relationship characteristics influence how empathic offenders respond to non‐forgiveness. For example, levels of relationship commitment are associated with relational repair behaviours (Rusbult et al., 2005). It could be that in high‐quality relationships (e.g., high relationship commitment), empathic offenders who receive non‐forgiveness could instead maintain or even increase their efforts at repairing the relationship because their concern for the relationship may outweigh their concern about their empathic response not being reciprocated.

Future research could investigate whether other traits or identities produce reciprocity expectations that enhance offenders' reactions to non‐forgiveness. For example, religious doctrines promote that people should seek forgiveness and should grant forgiveness, when wronged by others (Rye et al., 2000). It is possible that offenders who see forgiveness as important to religious practice may be more active in conflict resolution and towards seeking forgiveness (Lambert & Dollahite, 2006) but may also expect forgiveness in return and react even more negatively to victims' non‐forgiveness, consistent with our findings.

## CONCLUSION

Forgiveness only has meaning if victims have the right to withhold it. Yet, when victims refuse to forgive, offenders who believe that they have shown efforts towards repair tend to feel victimized. Paradoxically, this is made worse when offenders have high trait empathy, as they tend to hold the view that victims should reciprocate their repair efforts with an empathic response. For moral repair processes that promote empathy, this represents a conundrum.

## AUTHOR CONTRIBUTIONS

**Blake Quinney:** Conceptualization; investigation; writing – original draft; methodology; validation; visualization; writing – review and editing; software; formal analysis; data curation; resources; project administration. **Michael Wenzel:** Conceptualization; funding acquisition; writing – review and editing; supervision; methodology. **Michael Thai:** Conceptualization; writing – review and editing; methodology. **Tyler Okimoto:** Conceptualization; methodology; writing – review and editing. **Lydia Woodyatt:** Conceptualization; methodology; writing – review and editing.

## FUNDING INFORMATION

This research was supported by a grant from the Australian Research Council (Grant No. DP190102283).

## CONFLICT OF INTEREST STATEMENT

All authors declare no conflict of interest.

## Supporting information


Data S1.

## Data Availability

The data are openly available in OSF at https://osf.io/c7kmx/?view_only=8b837b5f7bad41bd8c2058b3c7553a5c.

## Footnotes

^1^
We had other variables and pre‐registered hypotheses for each study. However, we omit the results testing these hypotheses from the main text to maintain the focus on trait empathic concern. These further results and discussions are present in the Online Material.

^2^
We conducted an experiment to investigate whether offenders may feel less like ‘victims’ if they focused on the hurt the victim felt after the wrongdoing vs. how the victim felt when refusing forgiveness. However, our manipulation check indicated that our manipulation was unsuccessful, and so we omitted this study and instead solely focus on empathy as a trait measure.

^3^
We reanalysed the data in Study 3 by including perceived severity, perceived intentionality and perceived forgivability as covariates, but the inclusion of these variables did not affect the pattern of results.

## References

[bjso70001-bib-0001] Batson, C. D. (2023). Empathic concern: What it is and why it's important. Oxford University Press.

[bjso70001-bib-0002] Baumeister, R. F. , Stillwell, A. , & Wotman, S. R. (1990). Victim and perpetrator accounts of interpersonal conflict: Autobiographical narratives about anger. Journal of Personality and Social Psychology, 59(5), 994. 10.1037/0022-3514.59.5.994 2266485

[bjso70001-bib-0003] Blader, S. L. , & Chen, Y.‐R. (2012). Differentiating the effects of status and power: A justice perspective. Journal of Personality and Social Psychology, 102(5), 994–1014. 10.1037/a0026651 22229456

[bjso70001-bib-0004] Bloom, P. (2017). Against empathy: The case for rational compassion. Random House.

[bjso70001-bib-0005] Bohns, V. K. , & Flynn, F. J. (2021). Empathy and expectations of others' willingness to help. Personality and Individual Differences, 168, 110368. 10.1016/j.paid.2020.110368

[bjso70001-bib-0006] Bono, G. , McCullough, M. E. , & Root, L. M. (2008). Forgiveness, feeling connected to others, and well‐being: Two longitudinal studies. Personality and Social Psychology Bulletin, 34(2), 182–195. 10.1177/0146167207310025 18063835

[bjso70001-bib-0007] Cuff, B. M. , Brown, S. J. , Taylor, L. , & Howat, D. J. (2016). Empathy: A review of the concept. Emotion Review, 8(2), 144–153. 10.1177/1754073914558466

[bjso70001-bib-0008] Davis, M. H. (1983). Measuring individual differences in empathy: Evidence for a multidimensional approach. Journal of Personality and Social Psychology, 44(1), 113–126. 10.1037/0022-3514.44.1.113

[bjso70001-bib-0009] Decety, J. (2021). Why empathy is not a reliable source of information in moral decision making. Current Directions in Psychological Science, 30(5), 425–430. 10.1177/09637214211031943

[bjso70001-bib-0065] Depow, G. J. , & Inzlicht, M. (2025). How individual differences in empathy predict moments of empathy in everyday life. Personality and Social Psychology Bulletin. 10.1177/01461672251333823 PMC13310264 40437765

[bjso70001-bib-0010] Dhami, M. K. (2016). Effects of a victim's response to an offender's apology: When the victim becomes the bad guy. European Journal of Social Psychology, 46(1), 110–123. 10.1002/ejsp.2145

[bjso70001-bib-0011] Enright, R. D. (2019). Forgiveness is a choice: A step‐by‐step process for resolving anger and restoring hope. American Psychological Association.

[bjso70001-bib-0012] Exline, J. J. , & Baumeister, R. F. (2000). Expressing forgiveness and repentance: Benefits and barriers. In M. E. McCullough , K. I. Pargament , & C. E. Thoresen (Eds.), Forgiveness: Theory, research, and practice (pp. 133–155). The Guilford Press.

[bjso70001-bib-0013] Feeney, J. , & Fitzgerald, J. (2019). Attachment, conflict and relationship quality: Laboratory‐based and clinical insights. Current Opinion in Psychology, 25, 127–131. 10.1016/j.copsyc.2018.04.002 29753972

[bjso70001-bib-0014] Fehr, R. , & Gelfand, M. J. (2012). The forgiving organization: A multilevel model of forgiveness at work. Academy of Management Review, 37(4), 664–688. 10.5465/amr.2010.0497

[bjso70001-bib-0015] Foster, C. A. , & Rusbult, C. E. (1999). Injustice and powerseeking. Personality and Social Psychology Bulletin, 25(7), 834–849. 10.1177/0146167299025007006

[bjso70001-bib-0016] Frantz, C. M. , & Bennigson, C. (2005). Better late than early: The influence of timing on apology effectiveness. Journal of Experimental Social Psychology, 41(2), 201–207. 10.1016/j.jesp.2004.07.007

[bjso70001-bib-0017] Gabay, R. , Hameiri, B. , Rubel‐Lifschitz, T. , & Nadler, A. (2020). The tendency for interpersonal victimhood: The personality construct and its consequences. Personality and Individual Differences, 165, 110134. 10.1016/j.paid.2020.110134

[bjso70001-bib-0018] Harth, N. S. , Hornsey, M. J. , & Barlow, F. K. (2011). Emotional responses to rejection of gestures of intergroup reconciliation. Personality and Social Psychology Bulletin, 37(6), 815–829. 10.1177/0146167211400617 21402754

[bjso70001-bib-0019] Hayes, A. F. (2022). Introduction to mediation, moderation, and conditional process analysis: A regression‐based approach. The Guilford Press.

[bjso70001-bib-0020] Howell, A. J. , Turowski, J. B. , & Buro, K. (2012). Guilt, empathy, and apology. Personality and Individual Differences, 53(7), 917–922. 10.1016/j.paid.2012.06.021

[bjso70001-bib-0021] Hu, Y. , Fiedler, S. , & Weber, B. (2020). What drives the (un) empathic bystander to intervene? Insights from eye tracking. British Journal of Social Psychology, 59(3), 733–751. 10.1111/bjso.12354 31792985

[bjso70001-bib-0022] Jennings, D. J. , Worthington, E. L. , Van Tongeren, D. R. , Hook, J. N. , Davis, D. E. , Gartner, A. L. , Greer, C. L. , & Mosher, D. K. (2016). The transgressor's response to denied forgiveness. Journal of Psychology and Theology, 44, 16–27. 10.1177/009164711604400102

[bjso70001-bib-0023] Johnson, J. , & Lecci, L. (2020). Does empathy undermine justice? Moderating the impact of empathic concern for a white policeman on responses to police interracial violence. British Journal of Social Psychology, 59(3), 752–772. 10.1111/bjso.12347 31693225

[bjso70001-bib-0024] Johnson, J. , Lecci, L. , & Dovidio, J. F. (2024). White Americans' blame attributions and empathy towards black victims of police violence: How pejorative stereotypes ‘engulf the field’. British Journal of Social Psychology, 63(2), 936–955. 10.1111/bjso.12712 38131304

[bjso70001-bib-0025] Karremans, J. C. , & Smith, P. K. (2010). Having the power to forgive: When the experience of power increases interpersonal forgiveness. Personality and Social Psychology Bulletin, 36(8), 1010–1023. 10.1177/0146167210376761 20693385

[bjso70001-bib-0026] Klimecki, O. M. (2019). The role of empathy and compassion in conflict resolution. Emotion Review, 11(4), 310–325. 10.1177/1754073919838609

[bjso70001-bib-0027] Lambert, N. M. , & Dollahite, D. C. (2006). How religiosity helps couples prevent, resolve, and overcome marital conflict. Family Relations, 55(4), 439–449. 10.1111/j.1741-3729.2006.00413.x

[bjso70001-bib-0028] Leary, M. R. , Twenge, J. M. , & Quinlivan, E. (2006). Interpersonal rejection as a determinant of anger and aggression. Personality and Social Psychology Review, 10(2), 111–132. 10.1207/s15327957pspr1002_2 16768650

[bjso70001-bib-0029] Leunissen, J. M. , Sedikides, C. , & Wildschut, T. (2017). Why narcissists are unwilling to apologise: The role of empathy and guilt. European Journal of Personality, 31(4), 385–403. 10.1002/per.2110

[bjso70001-bib-0030] Litman, L. , Robinson, J. , & Abberbock, T. (2017). A versatile crowdsourcing data acquisition platform for the behavioral sciences. Behavior Research Methods, 49(2), 433–442. 10.3758/s13428-016-0727-z 27071389 PMC5405057

[bjso70001-bib-0031] McAuliffe, W. H. , Carter, E. C. , Berhane, J. , Snihur, A. C. , & McCullough, M. E. (2020). Is empathy the default response to suffering? A meta‐analytic evaluation of perspective taking's effect on empathic concern. Personality and Social Psychology Review, 24(2), 141–162. 10.1177/1088868319887599 31771425

[bjso70001-bib-0032] McCullough, M. E. , & Hoyt, W. T. (2002). Transgression‐related motivational dispositions: Personality substrates of forgiveness and their links to the big five. Personality and Social Psychology Bulletin, 28(11), 1556–1573. 10.1177/014616702237583

[bjso70001-bib-0033] Muller, D. , Judd, C. M. , & Yzerbyt, V. Y. (2005). When moderation is mediated and mediation is moderated. Journal of Personality and Social Psychology, 89(6), 852. 10.1037/0022-3514.89.6.852 16393020

[bjso70001-bib-0034] Muthén, B. , & Muthén, L. (2017). Mplus. In Handbook of item response theory (pp. 507–518). Chapman and Hall/CRC.

[bjso70001-bib-0035] Okimoto, T. G. , Wenzel, M. , & Hedrick, K. (2013). Refusing to apologise can have psychological benefits (and we issue no mea culpa for this research finding). European Journal of Social Psychology, 43(1), 22–31. 10.1002/ejsp.1901

[bjso70001-bib-0036] Okimoto, T. G. , Wenzel, M. , & Hornsey, M. J. (2015). Apologies demanded yet devalued: Normative dilution in the age of apology. Journal of Experimental Social Psychology, 60, 133–136. 10.1016/j.jesp.2015.05.008

[bjso70001-bib-0037] Paulhus, D. L. , & Vazire, S. (2007). The self‐report method. In R. W. Robins , R. C. Fraley , & R. F. Krueger (Eds.), Handbook of research methods in personality psychology (pp. 224–239). The Guilford Press.

[bjso70001-bib-0038] Pearce, H. , Strelan, P. , & Burns, N. R. (2018). The barriers to forgiveness scale: A measure of active and reactive reasons for withholding forgiveness. Personality and Individual Differences, 134, 337–347. 10.1016/j.paid.2018.06.042

[bjso70001-bib-0039] Pirlott, A. G. , & MacKinnon, D. P. (2016). Design approaches to experimental mediation. Journal of Experimental Social Psychology, 66, 29–38. 10.1016/j.jesp.2015.09.012 27570259 PMC4999253

[bjso70001-bib-0040] Quinney, B. , Wenzel, M. , & Woodyatt, L. (2024). The role of truth in victim–offender mediation: Victims of crime who feel they know the “whole” truth are more receptive to apologies. Law and Human Behavior, 48(3), 228–245. 10.1037/lhb0000564 38949768

[bjso70001-bib-0064] Quinney, B. , Wenzel, M. , Thai, M. , Okimoto, T. , & Woodyatt, L. (2024). Is it genuine or pseudo‐forgiveness? Offenders' appraisals of victims' expressed forgiveness as a function of engagement in co‐reflection. International Review of Social Psychology, 37(1), 15. 10.5334/irsp.887 PMC12372707 40950748

[bjso70001-bib-0041] Risen, J. L. , & Gilovich, T. (2007). Target and observer differences in the acceptance of questionable apologies. Journal of Personality and Social Psychology, 92(3), 418. 10.1037/0022-3514.92.3.418 17352601

[bjso70001-bib-0042] Rusbult, C. E. , Hannon, P. A. , Stocker, S. L. , & Finkel, E. J. (2005). Forgiveness and relational repair. Handbook of Forgiveness, 20, 185–205.

[bjso70001-bib-0043] Rye, M. S. , Pargament, K. I. , Ali, M. A. , Beck, G. L. , Dorff, E. N. , Hallisey, C. , Narayanan, V. , & Williams, J. G. (2000). Religious perspectives on forgiveness. In M. E. McCullough , K. I. Pargament , & C. E. Thoresen (Eds.), Forgiveness: Theory, research, and practice (pp. 17–40). The Guilford Press.

[bjso70001-bib-0044] Scher, S. J. , & Darley, J. M. (1997). How effective are the things people say to apologise? Effects of the realization of the apology speech act. Journal of Psycholinguistic Research, 26, 127–140. 10.1023/A:1025068306386

[bjso70001-bib-0045] Schumann, K. (2014). An affirmed self and a better apology: The effect of self‐affirmation on transgressors' responses to victims. Journal of Experimental Social Psychology, 54, 89–96. 10.1016/j.jesp.2014.04.013

[bjso70001-bib-0046] Schumann, K. , & Dragotta, A. (2021). Empathy as a predictor of high‐quality interpersonal apologies. European Journal of Social Psychology, 51(6), 896–909. 10.1002/ejsp.2786

[bjso70001-bib-0047] Shnabel, N. , Nadler, A. , Ullrich, J. , Dovidio, J. F. , & Carmi, D. (2009). Promoting reconciliation through the satisfaction of the emotional needs of victimised and perpetrating group members: The needs‐based model of reconciliation. Personality and Social Psychology Bulletin, 35(8), 1021–1030. 10.1177/0146167209336610 19498070

[bjso70001-bib-0048] SimanTov‐Nachlieli, I. , & Shnabel, N. (2014). Feeling both victim and perpetrator: Investigating duality within the needs‐based model. Personality and Social Psychology Bulletin, 40(3), 301–314. 10.1177/0146167213510746 24219990

[bjso70001-bib-0049] Simas, E. N. , Clifford, S. , & Kirkland, J. H. (2020). How empathic concern fuels political polarization. American Political Science Review, 114(1), 258–269. 10.1017/S0003055419000534

[bjso70001-bib-0063] Spencer, S. J. , Zanna, M. P. , & Fong, G. T. (2005). Establishing a causal chain: Why experiments are often more effective than mediational analyses in examining psychological processes. Journal of Personality and Social Psychology, 89(6), 845–851. 10.1037/0022-3514.89.6.845 16393019

[bjso70001-bib-0062] Takada, N. , & Ohbuchi, K. (2013). True and hollow forgiveness, forgiveness motives, and conflict resolution. International Journal of Conflict Management, 24(2), 184–200. 10.1108/10444061311316799

[bjso70001-bib-0050] Thai, M. , Wenzel, M. , & Okimoto, T. G. (2023). Turning tables: Offenders feel like “victims” when victims withhold forgiveness. Personality and Social Psychology Bulletin, 49(2), 233–250. 10.1177/014616722110624 34964377

[bjso70001-bib-0051] vanOyen‐Witvliet, C. , Ludwig, T. , & Bauer, D. J. (2002). Please forgive me: Transgressors' emotions and physiology during imagery of seeking forgiveness and victim responses. Journal of Psychology and Christianity, 21, 219.

[bjso70001-bib-0052] Wallis, P. (2014). Understanding restorative justice: How empathy can close the gap created by crime. Policy Press.

[bjso70001-bib-0053] Wenzel, M. , & Okimoto, T. G. (2010). How acts of forgiveness restore a sense of justice: Addressing status/power and value concerns raised by transgressions. European Journal of Social Psychology, 40(3), 401–417. 10.1002/ejsp.629

[bjso70001-bib-0054] Wenzel, M. , Woodyatt, L. , & Hedrick, K. (2012). No genuine self‐forgiveness without accepting responsibility: Value reaffirmation as a key to maintaining positive self‐ regard. European Journal of Social Psychology, 42(5), 617–627. 10.1002/ejsp.1873

[bjso70001-bib-0055] Wenzel, M. , Woodyatt, L. , Okimoto, T. G. , & Worthington, E. L., Jr. (2021). Dynamics of moral repair: Forgiveness, self‐forgiveness, and the restoration of value consensus as interdependent processes. Personality and Social Psychology Bulletin, 47(4), 607–626. 10.1177/01461672209375 32674663

[bjso70001-bib-0057] Wohl, M. J. , & McGrath, A. L. (2007). The perception of time heals all wounds: Temporal distance affects willingness to forgive following an interpersonal transgression. Personality and Social Psychology Bulletin, 33(7), 1023–1035. 10.1177/014616720730102 17554013

[bjso70001-bib-0056] Wohl, M. J. , Kuiken, D. , & Noels, K. A. (2006). Three ways to forgive: A numerically aided phenomenological study. British Journal of Social Psychology, 45(3), 547–561. 10.1348/014466605X53695 16984720

[bjso70001-bib-0058] Woodyatt, L. , & Wenzel, M. (2013). The psychological immune response in the face of transgressions: Pseudo self‐forgiveness and threat to belonging. Journal of Experimental Social Psychology, 49(6), 951–958. 10.1016/j.jesp.2013.05.016

[bjso70001-bib-0059] Woodyatt, L. , Wenzel, M. , Okimoto, T. G. , & Thai, M. (2022). Interpersonal transgressions and psychological loss: Understanding moral repair as dyadic, reciprocal, and interactionist. Current Opinion in Psychology, 44, 7–11. 10.1016/j.copsyc.2021.08.018 34534843

[bjso70001-bib-0060] Zaki, J. (2014). Empathy: A motivated account. Psychological Bulletin, 140(6), 1608–1647. 10.1037/a0037679 25347133

[bjso70001-bib-0061] Zaki, J. , & Cikara, M. (2015). Addressing empathic failures. Current Directions in Psychological Science, 24(6), 471–476. 10.1177/0963721415599978

