# Supplementary material for: Empathy expectations: Trait empathy exacerbates apologetic offenders' negative reactions to non‐forgiveness
Source: Br J Soc Psychol. 2025 Jun 24;64(3):e70001. doi: 10.1111/bjso.70001 (PMC12186105; doi:10.1111/bjso.70001)
Supplement: Supplementary file 1 — Data S1. [file BJSO-64-0-s001.docx]

**Supplementary Material**

The present research also examined whether offenders may denigrate non-forgiving victims. We thus had additional pre-registered predictions which were supported across all studies, but we report here to maintain the focus on empathy in the main manuscript. We predicted that participants in the non-forgiveness (versus forgiveness) would report lower victim’s moral character, greater victim deservingness, and lower willingness to reconcile with the victim. We also predicted that victim’s moral character, and victim deservingness, will mediate the effect of victimhood perception on willingness to reconcile. Specifically, victimhood perception will be negatively related to victim’s perceived moral character, which, in turn, will be positively related to willingness to reconcile. We also predicted that victimhood perception will be positively related to victim’s perceived deservingness of the initial harm, which, in turn, will be negatively related to willingness to reconcile.

We also made predictions that *fantasy* (a form of cognitive empathy) would moderate the effect of non-forgiveness on norm violation perceptions and threat to status/power. However, we do not report these results in the main text because these results were inconsistent, and like Davis (1994), we find the fantasy subscale difficult to interpret.

**Study 1**

**Method**

***Additional Measures***

**Victim Moral Character.** The nine-item scale from Philpot & Hornsey (2008) assessed participants’ evaluations of the victims’ moral character (e.g., “*This person is moral*”; 1 = not at all, 7 = very much; α = .95).

**Victim Deservingness.** The five-item scale from Reynolds et al. (2020) measured participants’ perception that the victim deserved the initial transgression (e.g., “*Perhaps this person deserved what happened*”; 1 = not at all, 7 = very much; α = .82).

**Willingness to Reconcile**. The eight-item scale from Onody et al. (2020) measured participants’ willingness to reconcile with the victim of their transgression (e.g., “*I would try harder to make amends with this person*”; α = .95).

**Results**

We conducted independent *t*-tests to analyse the effect of non-forgiveness (vs. forgiveness) on all dependent variables. Supplementary Table 1 presents the descriptive statistics, *t*-tests, and effect sizes. Consistent with hypotheses, participants (*N* = 147) in the non-forgiveness condition reported lower victim moral character, greater victim deservingness, and lower willingness to reconcile, than participants in the forgiveness condition (*N* = 150).

***Path Analyses***

We used IBM Amos v.29 to test the paths for our hypothesised model. Our initial model demonstrated support for most hypothesised paths, but poor fit to the data: χ^2^(11) = 131.4; CFI = .89; RMSEA = .19; SRMR = .13. The modification indices suggested additional theoretically viable paths including a direct path from non-forgiveness to willingness to reconcile, and direct paths from norm violation perceptions to victim’s moral character, and victim deservingness. The resulting model fit was improved: χ^2^(8) = 17.0; CFI = .99; RMSEA = .06; SRMR = .02. Supplementary Figure 1 presents the standardised coefficients for this model.

We tested the specific indirect effect pathways via bootstrapping methods. Supplementary Table 2 presents the results of these indirect effects analyses. We found a significant positive indirect effect of non-forgiveness (vs. forgiveness) on victimhood perceptions via increased norm violation perceptions, but no significant indirect effect via threat to status/power. There was also a significant negative indirect effect of victimhood perceptions on willingness to reconcile via decreased victim’s moral character, but no significant indirect effect via victim deservingness.

**Pre-Registered Moderation Analysis**

We used Process Model 7 (Hayes, 2017) to test our moderation analyses. Bootstraps were set at 5,000 replications to estimate 95% percentile confidence intervals. Conditional effects were calculated at -1SD and +1SD to represent low and high levels of the moderator, respectively. We predicted that psychological entitlement (Campbell et al., 2004) would moderate the effect of non-forgiveness (vs. forgiveness) on victimhood perceptions, via threat to status/power. However, the interaction between non-forgiveness (vs. forgiveness) and psychological entitlement was not significant, *B* = 0.07, *SE* = 0.08, *p* = .41.

***Exploratory Moderation Analyses***

We also used Process Model 7 (Hayes, 2017) for our exploratory moderation analyses. We explored whether empathic concern may also moderate the effect of non-forgiveness (vs. forgiveness) on threat to status/power (we included norm violation perceptions also as a mediator in this analysis). Indeed, empathic concern significantly strengthened the effect of non-forgiveness on threat to status/power. However, the index of moderated-mediation was non-significant. See Supplementary Table 3.

We conducted several further moderation analyses with the other subscales of the interpersonal reactivity index (Davis, 1980). We found that fantasy significantly moderated the effect of non-forgiveness (vs. forgiveness) on norm violation perceptions, and threat to status/power. However, the index of moderated-mediation with threat to status/power as the mediator was non-significant. The results for the moderating effect of fantasy on non-forgiveness (vs. forgiveness) on victimhood perceptions, via norm violation perceptions, and threat to status/power, are presented in Supplementary Table 4, and Supplementary Table 5, respectively. Perspective-taking did not significantly moderate the effect of non-forgiveness (vs. forgiveness) on norm violation perceptions (*B* = 0.15, *SE* = 0.09, *p* = .108), or threat to status/power (*B* = 0.16, *SE* = 0.08, *p* = .063). Personal distress also did not significantly moderate the effect of non-forgiveness (vs. forgiveness) on norm violation perceptions (*B* = -0.06, *SE* = 0.09, *p* = .557), or threat to status/power (*B* = 0.03, *SE* = 0.08, *p* = .693).

We also tested for moderation by *affective empathy*, the combination of the subscales empathic concern and personal distress. However, there was no significant interaction between non-forgiveness and affective empathy on norm violation perceptions, *B* = 0.13, *SE* = 0.09, *p* = .182, or threat to status/power, *B* = 0.09, *SE* = 0.08, *p* = .266. Finally, we tested for moderation by *cognitive empathy,* the combination of the subscales fantasy and perspective-taking. We observed a significant interaction between non-forgiveness and cognitive empathy on norm violation perceptions (*B* = 0.25, *SE* = 0.09, *p* = .007), and threat to status/power (*B* = 0.27, *SE* = 0.08, *p* = .001). We omit the full details of these moderation analyses given that they mirror the results of the fantasy moderation analyses.

**Study 2**

**Method**

***Measures***

We used the same scales from Study 1 to measure victim moral character (α = .94), victim deservingness (α = .80), and willingness to reconcile (α = .93).

**Results**

We conducted independent *t*-tests to analyse the effect of non-forgiveness (vs. forgiveness) on all dependent variables. Supplementary Table 6 presents the descriptive statistics, *t*-tests, and effect sizes. Consistent with hypotheses, participants (*N* = 150) in the non-forgiveness condition reported lower victim moral character, greater victim deservingness, and lower willingness to reconcile, than participants in the forgiveness condition (*N* = 146).

***Path Analyses***

We conducted path analyses to test our hypothesised model. Our initial model demonstrated support for the hypothesized paths, but poor fit to the data: χ^2^(10) = 151.9; CFI = .86; RMSEA = .21; SRMR = .14. The modification indices suggested including a direct path from non-forgiveness to willingness to reconcile, a direct path from norm violation perceptions to victim’s moral character, and direct paths from norm violation perceptions and threat to status/power to victim deservingness. The resulting model fit was improved: χ^2^(7) = 10.8; CFI = .99; RMSEA = .04; SRMR = .02. Supplementary Figure 2 presents the standardised coefficients for this model.

Supplementary Table 2 presents the results for the indirect effects analyses. There was a significant positive indirect effect of non-forgiveness (vs. forgiveness) on victimhood perceptions via increased norm violation perceptions, and a significant positive indirect effect via increased threat to status/power. There was also a significant negative indirect effect of victimhood perceptions on willingness to reconcile via decreased victim’s moral character, but no significant indirect effect via victim deservingness.

**Pre-Registered Moderation Analysis**

We used Process Model 7 (Hayes, 2017) to test for moderated-mediation with bootstraps set at 5,000 replications to estimate 95% percentile confidence intervals. Again, conditional effects were calculated at -1SD and +1SD to represent low and high levels of the moderator. We predicted that fantasy would moderate the effect of non-forgiveness (vs. forgiveness) on victimhood perceptions via norm violation perceptions. However, there was no significant interaction between non-forgiveness and fantasy on norm violation perceptions (*B* = 0.14, *SE* = 0.10, *p* = .155).

***Exploratory Moderation Analyses***

We also explored whether emotional reactivity may moderate the effect of non-forgiveness on norm violation perceptions, but this was not significant: *B* = 0.04, *SE* = 0.10, *p* = .72. However, general norm of reciprocity did moderate the effect of non-forgiveness on norm violation perceptions and Supplementary Table 7 presents these results. However, the index of moderated-mediation was non-significant.

**Study 3**

**Method**

***Measures***

We used the same scales to measure victim moral character (α = .95), victim deservingness (α = .78), and willingness to reconcile (α = .93).

**Results**

We conducted independent *t*-tests to analyse the effect of non-forgiveness (vs. forgiveness) on all dependent variables. Supplementary Table 8 presents the descriptive statistics, *t*-tests, and effect sizes. Consistent with hypotheses, participants (*N* = 203) in the non-forgiveness condition reported lower victim moral character, greater victim deservingness, and lower willingness to reconcile, than participants in the forgiveness condition (*N* = 204).

***Path Analyses***

We conducted path analyses to test our hypothesized model. Our initial model demonstrated support for the hypothesized paths, but poor fit to the data: χ^2^(11) = 221.3; CFI = .86; RMSEA = .22; SRMR = .14. The modification indices suggested additional theoretically viable paths including a direct path from non-forgiveness to willingness to reconcile, direct paths from norm violation perceptions to victim deservingness and victim’s moral character, and a direct path from threat to status/power to victim deservingness. The resulting model fit was improved: χ^2^(7) = 19.3; CFI = .99; RMSEA = .07; SRMR = .03. Figure 3 presents the standardized coefficients for this model.

Supplementary Table 2 presents the results for the indirect effects testing. There were significant positive indirect effects of non-forgiveness (vs. forgiveness) on victimhood perceptions via increased norm violation perceptions, and via increased threat to status/power. There were also significant negative indirect effects of victimhood perceptions on willingness to reconcile via decreased victim’s moral character, and increased victim deservingness.

**Pre-Registered Moderation Analysis**

We used Process Model 7 (Hayes, 2017) to test our moderated-mediation analyses. We predicted that fantasy would moderate the effect of non-forgiveness (vs. forgiveness) on victimhood perceptions via norm violation perceptions, and threat to status/power. There was no significant interaction between non-forgiveness and fantasy on norm violation perceptions, *B* = 0.08, *SE* = 0.04, *p* = .05; index of moderated-mediation: *B* = 0.04, *SE* = 0.02, 95%CI [-0.01, 0.89]. However, we found that fantasy significantly moderated the effect of non-forgiveness (vs. forgiveness) on threat to status/power (see Supplementary Table 9).

|  | Victim Response to Apology | |  |  |
| --- | --- | --- | --- | --- |
| Variable | Forgiveness  *M* (*SD*) | Non-Forgiveness  *M* (*SD*) | *t*(295) | *d* [95% CI] |
| 1. Victim’s Moral Character | 6.19 (0.84) | 5.06 (1.44) | 8.26 | 0.96 [0.72, 1.20] |
| 2. Victim Deservingness | 2.53 (1.25) | 3.09 (1.49) | -3.52 | 0.41 [0.18, 0.64] |
| 3. Willingness to Reconcile | 6.08 (0.88) | 4.66 (1.64) | 9.30 | 1.08 [0.84, 1.32] |

**Supplementary Table 1.** Descriptive and Inferential Statistics for Additional Variables (Study 1).

*Note.* All *p* values are significant at < .001.

**Supplementary Table 2.** Bootstrapped Indirect Effects Across All Studies.

| Pathways | *IE* | *SE* | *p* | 95% CI |
| --- | --- | --- | --- | --- |
| *Study 1*  **Non-Forgiveness 🡪 Norm Violation Perceptions 🡪 Victimhood Perceptions** | **1.21** | **0.15** | **< .001** | **0.92, 1.53** |
| Non-Forgiveness 🡪 Threat to status/power 🡪 Victimhood Perceptions | 0.04 | 0.13 | .806 | -0.22, 0.29 |
| **Victimhood Perceptions 🡪 Victim’s Moral Character 🡪 Willingness to Reconcile** | **-0.15** | **0.04** | **< .001** | **-0.24, -0.07** |
| Victimhood Perceptions 🡪 Victim Deservingness 🡪 Willingness to Reconcile | -0.02 | 0.02 | .252 | -0.05, 0.01 |
| *Study 2*  **Non-Forgiveness 🡪 Norm Violation Perceptions 🡪 Victimhood Perceptions** | **0.93** | **0.15** | **< .001** | **0.65, 1.22** |
| **Non-Forgiveness 🡪 Threat to status/power 🡪 Victimhood Perceptions** | **0.72** | **0.13** | **< .001** | **0.46, 0.98** |
| **Victimhood Perceptions 🡪 Victim’s Moral Character 🡪 Willingness to Reconcile** | **-0.08** | **0.03** | **.001** | **-0.13, -0.03** |
| Victimhood Perceptions 🡪 Victim Deservingness 🡪 Willingness to Reconcile  *Study 3* | -0.01 | 0.01 | .104 | -0.03, 0.01 |
| **Non-Forgiveness 🡪 Norm Violation Perceptions 🡪 Victimhood Perceptions** | **1.19** | **0.13** | **< .001** | **0.95, 1.44** |
| **Non-Forgiveness 🡪 Threat to status/power 🡪 Victimhood Perceptions** | **0.35** | **0.11** | **< .001** | **0.15, 0.57** |
| **Victimhood Perceptions 🡪 Victim’s Moral Character 🡪 Willingness to Reconcile** | **-0.09** | **0.03** | **= .001** | **-0.15, -0.04** |
| **Victimhood Perceptions 🡪 Victim Deservingness 🡪 Willingness to Reconcile** | **-0.01** | **0.01** | **= .022** | **-0.03, -0.01** |

*Note.* 5,000 bias-corrected bootstrapped samples calculated 95% confidence intervals. Bolded indirect effects are statistically significant.

| **Supplementary Table 3.** Moderated-Mediation of Empathic Concern on the effect of Non-Forgiveness on Victimhood perceptions via Threat to status/power (Study 1). | | | | | |
| --- | --- | --- | --- | --- | --- |
| *Variable* |  | *B* | SE *B* | *p* | CI_95%_ |
| *Threat to status/power* |  |  | *R* = .70, *R^2^* = .50, *p* < .001 | | |
| Constant |  | -2.06 | 0.13 | < .001 | [-2.32, 1.81] |
| Non-Forgiveness |  | 1.39 | 0.08 | < .001 | [1.23, 1.55] |
| Empathic Concern |  | -0.32 | 0.13 | .018 | [-0.58, -0.05] |
| Non-Forgiveness x Empathic Concern | | 0.21 | 0.08 | .013 | [0.04, 037] |
|  |  |  |  |  |  |
| High Empathic Concern (Simple Slope) | | 1.60 | 0.12 | < .001 | [1.37, 1.83] |
| Low Empathic Concern (Simple Slope) | | 1.18 | 0.12 | < .001 | [0.95, 1.41] |
|  | |  |  |  |  |
| *Victimhood perceptions* |  |  | *R* = .62, *R^2^* = .38, *p* < .001 | | |
| Constant |  | 0.39 | 0.21 | .069 | [-0.03, 0.81] |
| Non-Forgiveness |  | -0.26 | 0.14 | .063 | [-0.54, 0.01] |
| Threat to status/power |  | 0.09 | 0.14 | .189 | [-0.04, 0.21] |
| Index of Moderated Mediation | | 0.02 | 0.02 | - | [-0.02, 0.06] |
|  |  |  |  |  |  |

| **Supplementary Table 4.** Moderated-Mediation of Fantasy on the effect of Non-Forgiveness on Victimhood perceptions via Norm Violation Perceptions (Study 1). | | | | | |
| --- | --- | --- | --- | --- | --- |
| *Variable* |  | *B* | SE *B* | *p* | CI_95%_ |
| *Norm Violation Perceptions* |  |  | *R* = .61, *R^2^* = .37, *p* < .001 | | |
| Constant |  | -1.76 | 0.15 | < .001 | [-2.05, -1.47] |
| Non-Forgiveness |  | 1.19 | 0.09 | < .001 | [1.00, 1.37] |
| Fantasy |  | -0.38 | 0.15 | .011 | [-0.67, -0.09] |
| Non-Forgiveness x Fantasy | | 0.25 | 0.09 | .008 | [0.07, 0.43] |
|  |  |  |  |  |  |
| High Fantasy (Simple Slope) | | 1.44 | 0.13 | < .001 | [1.18, 1.70] |
| Low Fantasy (Simple Slope) | | 0.94 | 0.13 | < .001 | [0.68, 1.20] |
|  | |  |  |  |  |
| *Victimhood perceptions* |  |  | *R* = .62, *R^2^* = .38, *p* < .001 | | |
| Constant |  | 0.39 | 0.21 | .069 | [-0.03, 0.81] |
| Non-Forgiveness |  | -0.26 | 0.14 | .063 | [-0.54, 0.01] |
| Norm Violation Perceptions |  | 0.65 | 0.06 | < .001 | [0.53, 0.76] |
| Index of Moderated Mediation | | 0.16 | 0.07 | - | [0.04, 0.30] |
|  |  |  |  |  |  |
| High Fantasy (Conditional Indirect Effect) | | 0.93 | 0.14 | - | [0.68, 1.22] |
| Low Fantasy (Conditional Indirect Effect) | | 0.61 | 0.10 | - | [0.42, 0.80] |
|  | |  |  |  |  |

| **Supplementary Table 5.** Moderated-Mediation of Fantasy on the effect of Non-Forgiveness on Victimhood perceptions via Threat to status/power (Study 1). | | | | | |
| --- | --- | --- | --- | --- | --- |
| *Variable* |  | *B* | SE *B* | *p* | CI_95%_ |
| *Threat to status/power* |  |  | *R* = .71, *R^2^* = .50, *p* < .001 | | |
| Constant |  | -2.06 | 0.13 | < .001 | [-2.32, -1.81] |
| Non-Forgiveness |  | 1.39 | 0.08 | < .001 | [1.23, 1.55] |
| Fantasy |  | -0.39 | 0.13 | .004 | [-0.65, -0.13] |
| Non-Forgiveness x Fantasy | | 0.26 | 0.08 | .002 | [0.10, 0.42] |
|  |  |  |  |  |  |
| High Fantasy (Simple Slope) | | 1.65 | 0.12 | < .001 | [1.42, 1.88] |
| Low Fantasy (Simple Slope) | | 1.13 | 0.12 | < .001 | [0.90, 1.36] |
|  | |  |  |  |  |
| *Victimhood perceptions* |  |  | *R* = .62, *R^2^* = .38, *p* < .001 | | |
| Constant |  | 0.39 | 0.21 | .069 | [-0.03, 0.81] |
| Non-Forgiveness |  | -0.26 | 0.14 | .063 | [-0.54, 0.01] |
| Threat to status/power |  | 0.09 | 0.06 | .189 | [-0.04, 0.21] |
| Index of Moderated Mediation | | 0.02 | 0.02 | - | [-0.01, 0.39] |
|  |  |  |  |  |  |

| **Supplementary Table 6.** Descriptive and Inferential Statistics for Additional Variables in Study 2 | | | | |
| --- | --- | --- | --- | --- |
|  | Victim Response to Apology | |  |  |
| Variable | Forgiveness  *M* (*SD*) | Non-Forgiveness  *M* (*SD*) | *t*(294) | *d* [95% CI] |
| 1. Victim’s Moral Character | 6.08 (0.87) | 4.95 (1.25) | 9.04 | 1.05 [0.81, 1.29] |
| 2. Victim Deservingness | 2.58 (1.24) | 3.20 (1.03) | -4.17 | 0.48 [0.25, 0.72] |
| 3. Willingness to Reconcile | 5.86 (1.03) | 4.66 (1.43) | 8.28 | 0.96 [0.72, 1.20] |

*Note.* All *p* values are significant at < .001.

| **Supplementary Table 7.** Moderated-Mediation of Personal Norm of Reciprocity (PNR) on the effect of Non-Forgiveness on Victimhood perceptions via Norm Violation Perceptions (Study 2). | | | | | |
| --- | --- | --- | --- | --- | --- |
| *Variable* |  | *B* | SE *B* | *p* | CI_95%_ |
| *Norm Violation Perceptions* |  |  | *R* = .55, *R^2^* = .31, *p* < .001 | | |
| Constant |  | -1.63 | 0.15 | < .001 | [-1.94, -1.33] |
| Non-Forgiveness |  | 1.09 | 0.09 | < .001 | [0.89, 1.28] |
| PNR |  | -0.25 | 0.15 | .109 | [-0.57, 0.06] |
| Non-Forgiveness x PNR | | 0.19 | 0.10 | .048 | [0.01, 0.39] |
|  |  |  |  |  |  |
| High PNR (Simple Slope) | | 1.28 | 0.14 | < .001 | [1.00, 1.55] |
| Low PNR (Simple Slope) | | 0.89 | 0.14 | < .001 | [0.62, 1.16] |
|  | |  |  |  |  |
| *Victimhood perceptions* |  |  | *R* = .65, *R^2^* = .42, *p* < .001 | | |
| Constant |  | 0.32 | 0.18 | .078 | [-0.04, 0.68] |
| Non-Forgiveness |  | -0.22 | 0.12 | .069 | [-0.45, 0.02] |
| Norm Violation Perceptions |  | 0.47 | 0.05 | < .001 | [0.36, 0.58] |
| Index of Moderated Mediation | | 0.09 | 0.05 | - | [-0.01, 0.19] |
|  |  |  |  |  |  |

| **Supplementary Table 8.** Descriptive and Inferential Statistics for Additional Variables in Study 3 | | | | |
| --- | --- | --- | --- | --- |
|  | Victim Response to Apology | |  |  |
| Variable | Forgiveness  *M* (*SD*) | Non-Forgiveness  *M* (*SD*) | *t*(405) | *d* [95% CI] |
| 1. Victim’s Moral Character | 6.20 (0.74) | 4.91 (1.08) | 12.4 | 1.23 [1.01, 1.44] |
| 2. Victim Deservingness | 2.48 (1.08) | 3.21 (1.24) | -6.38 | 0.63 [0.43, 0.83] |
| 3. Willingness to Reconcile | 5.91 (0.89) | 4.55 (1.47) | 11.3 | 1.12 [0.91, 1.33] |

*Note.* All *p* values are significant at < .001.

| **Supplementary Table 9.** Moderated-Mediation of Fantasy on the effect of Non-Forgiveness on Victimhood perceptions via Threat to status/power (Study 3). | | | | | |
| --- | --- | --- | --- | --- | --- |
| *Variable* |  | *B* | SE *B* | *p* | CI_95%_ |
| *Threat to status/power* |  |  | *R* = .65, *R^2^* = .43, *p* < .001 | | |
| Constant |  | -0.01 | 0.04 | .998 | [-0.07, 0.07] |
| Non-Forgiveness |  | 0.65 | 0.04 | < .001 | [0.57, 0.72] |
| Fantasy |  | 0.03 | 0.04 | .452 | [-0.05, 0.10] |
| Non-Forgiveness x Fantasy | | 0.08 | 0.04 | .036 | [0.01, 0.15] |
|  |  |  |  |  |  |
| High Fantasy (Simple Slope) | | 0.72 | 0.05 | < .001 | [0.62, 0.83] |
| Low Fantasy (Simple Slope) | | 0.57 | 0.05 | < .001 | [0.46, 0.67] |
|  | |  |  |  |  |
| *Victimhood perceptions* |  |  | *R* = .65, *R^2^* = .42, *p* < .001 | | |
| Constant |  | -0.01 | 0.04 | .996 | [-0.07, 0.07] |
| Non-Forgiveness |  | -0.07 | 0.06 | .187 | [-0.18, 0.04] |
| Threat to status/power |  | 0.19 | 0.05 | < .001 | [0.09, 0.29] |
| Index of Moderated Mediation | | 0.01 | 0.01 | - | [0.01, 0.03] |
|  |  |  |  |  |  |
| High Fantasy (Conditional Indirect Effect) | | 0.14 | 0.04 | - | [0.06, 0.22] |
| Low Fantasy (Conditional Indirect Effect) | | 0.11 | 0.03 | - | [0.05, 0.18] |
|  | |  |  |  |  |

**Supplementary Figure 1**

*Final path model with standardized coefficients linking non-forgiveness to willingness to reconcile (Study 1).*

-.17 ^***^

-.54^***^

Victim Moral Character

Norm Violation Perceptions

-.25^***^

.60^***^

.68^***^

.60^***^

Non-Forgiveness (vs. Forgiveness)

Victimhood perceptions

Willingness to Reconcile

Empathic Concern

Threat to status/power

Victim Deservingness

.02

.26^**^

.22^***^

.34^***^

-.05

.70^***^

.21^*^

**Supplementary Figure 2**

*Final path model with standardized coefficients linking non-forgiveness to willingness to reconcile (Study 2)*

-.10^*^

-.62^***^

Victim Moral Character

Norm Violation Perceptions

-.17^***^

.54^***^

.67^***^

.43^***^

Non-Forgiveness (vs. Forgiveness)

Victimhood perceptions

Willingness to Reconcile

Empathic Concern

Threat to status/power

Victim Deservingness

.31^***^

.40^***^

.16^*^

.11

-.10^*^

.59^***^

-.16^**^

.34^*^

**Supplementary Figure 3**

*Final path model with standardized coefficients linking non-forgiveness to willingness to reconcile (Study 3)*

-.15^***^

-.62^***^

Victim Moral Character

Norm Violation Perceptions

-.19^***^

.62^***^

.59^***^

.55^***^

Non-Forgiveness (vs. Forgiveness)

Victimhood perceptions

Willingness to Reconcile

.15^***^

Threat to status/power

Victim Deservingness

.16^***^

.06

.03

.65^***^

.40^***^

.16^**^

-.10^**^

.09^*^

-.16^**^

Reciprocity Expectation

Empathic Concern
